# Molecular identification of *Fusarium* species complex isolated from clinical samples and its antifungal susceptibility patterns 

**DOI:** 10.18502/cmm.5.4.2149

**Published:** 2019

**Authors:** Yashik Bansal, Nidhi Singla, Neelam Kaistha, Sunandan Sood, Jagdish Chander

**Affiliations:** 1 Department of Microbiology, Government Medical College Hospital, Chandigarh, India; 2 Department of Ophthalmology, Government Medical College Hospital, Chandigarh, India

**Keywords:** Fusarium, Molecular identification, Keratitis, Onychomycosis, Taxonomy

## Abstract

**Background and Purpose:**

More than 300 *Fusarium* species are grouped into approximately 23 species complexes out of which around 70 are involved in human infections. The nomenclature of these species has undergone considerable changes in recent years. These species cause localized infections in individuals while inducing systemic infections mainly in immunocompromised patients. The present study was conducted to identify *Fusarium* species in clinical isolates by molecular methods and determine their in vitro minimum inhibitory concentration (MIC) patterns to address the lack of data in this domain in Northern India.

**Materials and Methods:**

For the purpose of the study, *Fusarium* isolates obtained from various clinical samples were sent to the Westerdijk Fungal Biodiversity Institute, Utrecht, the Netherlands, for molecular identification. The MIC testing was performed using the microbroth dilution method as per the Clinical and Laboratory Standards Institute reference method (M38-A2).

**Results:**

*Fusarium* was isolated from 33 patients (i.e., 1, 1, 2, 14, and 15 cases with endophthalmitis, sinusitis, pulmonary involvement, onychomycosis, and keratitis, respectively). These 33 isolates belonged to three species complexes, namely *F. solani* species complex (FSSC; n=13), *F. fujikuroi* species complex (FFSC; n=13), and *F. incarnatum* *equiseti* species complex (FIESC; n=7). The species identified within FSSC, FFSC, and FIESC included *F. keratoplasticum* (n=6)/*F. falciforme* (n=6)/*F. solani* (n=1), *F. proliferatum* (n=7)/*F. sacchari* (n=5)/*F. anthophilum* (n=1), and *F. incarnatum *SC species (n=6)/*F. equiseti* SC species (n=1), respectively. The MIC results showed that all isolates had a lower MIC against amphotericin B than against the other antifungal agents.

## Conclusion:

Timely diagnosis and appropriate treatment will facilitate the improvement of patient outcomes.

## Introduction

The fungi belonging to the genus *Fusarium* possess such attributes as the capability to grow on a wide range of substrates and present as biofilms [1] on water and in plumbing systems, setting the ground for the widespread distribution of this fungus [2, 3]. These species are well-known plant pathogens [4] that account for the contamination and spoilage of food [5]. More than 300 *Fusarium* species [6] have been identified, out of which around 70 species are involved in human infections, causing fusariosis [7]. The incidence of fungal infections in humans is increasing with the rising population of immunocompromised individuals [8, 9]. Accordingly, there is a proportional increase in the morbidity and mortality caused by fusariosis [10].

*Fusarium* used to have a dual nomenclature based on different ascomycete teleomorphs (i.e., *Gibberella, Nectria, Neocosmospora*, *Haematonectria*, *Cyanonectria*, *Geejayessia,* and *Albonectria*) [11, 12]. In January 2013, the International Code of Nomenclature for algae, fungi, and plants prohibited the dual nomenclature system. Therefore, the genus *Fusarium* was recognized as the standard name because it was the most commonly used name in the literature [13]. However, during the 2017 event at Shenzhen, China, the 19^th^ International Botanical Congress reversed its Article 57.2 and allowed to use the competing asexual and sexual names in the literature [14]. Still, the nomenclature has undergone substantial changes in recent years [7]. *Fusarium* species are now grouped into 23 grossly phylogenetic species complexes [6].

The species reported to be most commonly associated with human infections are *F. solani* (60%) and *F. oxysporum* (20%) [2]. However, these two species have been elevated to species complex status, and newer species have been identified and described recently [7]. Some of these species complexes that are commonly implicated in human infections include *F. solani* species complex (FSSC), *F. fujikuroi* species complex (FFSC), *F. incarnatum-equiseti* species complex (FIESC), *F. oxysporum* species complex (FOSC), *F. dimerum* species complex (FDSC), *F. chlamydosporum* species complex (FCSC), and *F. tricinctum* species complex (FTSC) [7].

The majority of *Fusarium* infections in immunocompetent individuals are superficial and subcutaneous, while systemic or disseminated infections are seen in immunocompromised individuals [4, 15]. Although *Fusarium* can be identified fairly well morphologically, molecular tests facilitate the accurate identification of the species and their classification in proper species complex [16]. Most of *Fusarium* species invariably show very high resistance to various antifungal agents. Accordingly, there is a species-specific resistance pattern to a particular antifungal agent [17, 18]. Amphotericin B is the only antifungal drug found to be consistently active during in vitro studies on various *Fusarium* species causing human infections [19].

In such a background of increasing fusariosis, susceptible host (immunocompromised individuals), changing nomenclature, taxonomy, and high in vitro antifungal resistance, the present study was conducted in a tertiary care center in North India to address the lack of data in this domain in this region.

## Materials and Methods

The present study was conducted on all the samples received in the Mycology Laboratory of the Department of Microbiology wherein *Fusarium* species had been isolated over a period of 24 months (i.e., October 2012 till October 2014). The obtained samples included corneal scrapings, donor sclera, recipient cornea, vitreous and aqueous tap, nail clippings, nasal polyps, sputum, and pleural or other body fluids.

The clinical samples were processed in the Department of Microbiology using the direct KOH/Calcofluor white mount examination, fungal culture, and antifungal susceptibility testing. The KOH and Calcofluor white mount (fluorescent microscopy) were performed to demonstrate the fungal elements in the clinical specimens. In addition, fungal culture was accomplished based on the standard mycological techniques using paired culture tubes of Sabouraud dextrose agar (SDA) with antibiotics (with chloramphenicol and gentamicin, without cycloheximide) and two tubes without antibiotics. One tube of each type was incubated at 37°C and 25°C. Subsequently, the fungal isolates on culture were identified at the genus level by conventional techniques, such as lactophenol cotton blue (LCB) mount [20]. Slide cultures of the isolates were put up whenever needed.

For species identification, the fungal isolates were sent to the Westerdijk Fungal Biodiversity Institute for molecular identification at species level. To this end, the DNAs of the clinical isolates were extracted and amplified by polymerase chain reaction (PCR) and sequenced as described before [21]. Genus identification and amplification were performed using ITS1 (5’-TCCGTAGGTGAACCTGCGG-3’)/ITS4 (5’-TCCTCCGCTTATTGATATGC-3’) or ITS5 (5’-GAAGTAAAAGTCGTAACAAGG-3’) and NL4 (5’-GGTCCGTGTTTCAAGACGG-3’) under standard conditions.

Species level identification was carried out via the multilocus sequence analysis using elongation factor 1-alpha and the DNA-directed RNA polymerase second largest subunit [21]. The PCR condition included [22] predenaturation for 3 min at 95°C, five cycles of 45 sec at 95°C, 45 sec at 58°C, and 2 min at 72°C, five cycles of 45 sec at 95°C, 45 sec at 56°C, and 2 min at 72°C, 30 cycles of 45 sec at 95°C, 45 sec at 52°C, and 2 min at 72°C, and a postelongation step of 8 min at 72°C.

Amplicon purification was accomplished using the Sephadex G-50 fine (HE Healthcare, Uppsala, Sweden). The amplicons were subjected to direct sequencing with the ABI Prism BIGDYE terminator cycle sequence kit (Applied Biosystems, Foster City, CA, USA). The ABI Prism 3730XL Sequencer was adopted to perform the final analysis. For identification purposes, the isolates were compared to the sequences in the FUSARIUM MLST database (http://www.cbs.knaw.nl/fusarium/) and Gen-Bank (http://blast.ncbi.nlm.nih.gov/Blast.cgi).

Antifungal susceptibility testing was carried out by broth microdilution method as per the Clinical Laboratory Standards Institute (CLSI) Reference Method for Broth Dilution Antifungal Susceptibility Testing of Filamentous Fungi, Approved Standard-Second Edition (M38-A2) [23]. The tested antifungals (i.e. amphotericin-B, voriconazole, itraconazole, fluconazole, caspofungin, and anidulafungin) were commercially sourced as powders from the Sigma-Aldrich.

Non-germinated conidial suspensions prepared by a spectrophotometric procedure and conidial test inocula in a range of approximately 0.4×10^4 ^to 5×10^4^ produced reproducible minimum inhibitory concentration (MIC). For inoculum preparation, conidia formation was achieved by growing *Fusarium* isolates on potato dextrose agar for up to 7 days. A suspension was then prepared in 0.85% normal saline, and conidia were separated from the hyphae by vortexing these suspension tubes. 

In the next stage, the supernatant containing the conidia was separated and subjected to spectrophotometry to measure the optical density at 530 nm that ranged from 0.15 to 0.17. The measured amount of saline or inoculum was added to adjust the optical density to the desired value. The proportion of inoculum and water added was noted, and the conidial suspension was prepared in RPMI-1640 using the same proportions. Finally, the RPMI suspension was diluted (1:50) by adding 4.9 ml of RPMI and 10 µl of the final inoculum.

Antifungal dilutions were prepared in RPMI for water-soluble and water-insoluble drugs as described in the CLSI document M38-A2 [15, 23]. To this end, in a single 96-well (i.e., 8 rows and 12 columns) “U” bottom sterile microtitre plate, 100 µl of each prepared drug dilution was added vertically to all wells in one column for each dilution. Then, 100 µl of the 1:50 diluted inoculum was added from well 1 to 10 horizontally in one row per fungal strain. Therefore, a total of 8 strains were tested per plate. Growth control (i.e., well containing only inoculum) and media control (i.e., drug-free PRMI-1640 medium) were put up in each plate as well. These plates were then covered with a lid and incubated at 35°C for 48 h. Finally, the plates were read visually using a reading mirror at the end of 48 h. The control strains used for internal quality control included ATCC 204304 *Aspergillus flavus* strain.

The study was conducted after obtaining ethical approval from the Ethical Clearance Committee of the Institute. Our research was based on ethical guidelines for biomedical research on human subjects based on the Central Ethics Committee on Human Research (CECHR) of Indian Council of Medical Research (ICMR), New Delhi, India, in 2006, and according to the ‘Declaration of Helsinki’ of 2008 [24, 25].

## Results

During the study period, a total of 1,905 samples were collected in the laboratory out of which 487 cases were positive for various fungal isolates. *Fusariosis* had a frequency of 6.8% among the positive samples with 33 samples yielding the growth of *Fusarium* species. The mean age of the patients was 45.4 years (range: 7-72 years). The number of male patients (n=24) was higher than that of female patients (n=9). Most of the patients presented with mycotic keratitis (n=15; 45.4%), followed by onychomycosis (n=14; 42.4%). Furthermore, there was one case of postoperative endophthalmitis and one case of sinusitis. Additionally, two patients who were immunocompromised had pulmonary involvement.

The obtained *Fusarium *species complexes were FSSC (n=13; 39.4%, including *F. keratoplasticum* [n=6], *F. falciforme* [n=6], and *F. solani* [n=1]), FFSC (n=13; 39.4%, including *F. proliferatum* [n=7], *F. sacchari* [n=5], and *F. anthophilum* [n=1]), and FIESC (n=7; 21.2%, including *F. incarnatum *SC species [n=6] and *F. equiseti* SC species [n=1]; Table 1).

As the majority of the patients had a superficial or subcutaneous involvement and only four patients had systemic involvement, the patients with superficial involvement were divided into two groups, namely keratitis (n=15) and onychomycosis (n=14) groups. The rest of the four cases were dealt with separately after these two groups.

With regard to the keratitis group, the mean age of the patients was 43.3 years (range: 7-70 years), and 14/15 (93.3%) patients were within the age group of 21-60 years. Out of these 15 patients, 9 cases were from the adjacent districts of Haryana, while 1, 1, and 4 cases were from Chandigarh, Uttar Pradesh, and Punjab, respectively. The median duration of symptoms was 20 days (mean: 22 days, range: 6-60 days). The *Fusarium* species complexes and species identified in these patients were FSSC (*F. falciforme *[n=5]*, F. keratoplasticum* [n=2], and *F. solani* [n=1]), FFSC (*F. sacchari* [n=4] and *F. proliferatum* [n=1]), and FIESC (*F. incarnatum* SC species [n=1] and *F. equiseti* SC species [n=1]).

In the onychomycosis group, the mean age of the patients was 47.4 years (range: 28-67 years). In addition, the median duration of symptoms was 12 months (range: 5-60 months). Out of these 14 patients, 13 cases were from in and around Chandigarh region. The *Fusarium* species complexes identified in these patients were FFSC (*F. proliferatum* [n=5], *F. sacchari* [n=1], and *F. anthophilum* [n=1]), FSSC (*F. keratoplasticum* [n=4]), and FIESC (*F. incarnatum* SC species. [n=3]). The risk factors identified in the two mentioned groups are shown in Table 2. 

There were a total of four other cases of fusariosis that are discussed below. The MIC values of these isolates were determined by the microbroth dilution method and reported for each antifungal agent as no breakpoints have been defined yet for *Fusarium* species in the literature. Table 3 presents the MIC of the isolates in this study.

**Table 1:** *Fusarium* species complexes and species isolated from 33 patients

**Species complex**	**Species**	**Clinical presentation (number)**
*F. solani* species complex	*Fusarium keratoplasticum*	Keratitis (n=2) Onychomycosis (n=4)
	*Fusarium falciforme*	Keratitis (n=5) Sinusitis (n=1)
	*Fusarium solani*	Keratitis (n=1)
*F. fujikuroi* species complex	*Fusarium proliferatum*	Keratitis (n=1) Onychomycosis (n=5) Pulmonary (n=1)
	*Fusarium sacchari*	Keratitis (n=4) Onychomycosis (n=1)
	*Fusarium anthophilum*	Onychomycosis (n=1)
*F. incarnatum* *equiseti* species complex	*Fusarium incarnatum *SC* sp*	Keratitis (n=1) Onychomycosis (n=3) Post-operative endophthalmitis (n=1) Pulmonary (n=1)
	*Fusarium equiseti *SC* sp*	Keratitis (n=1)

**Table 2:** Risk factors observed in patients with keratitis and onychomycosis

**Keratitis patients (n=15)**	**Onychomycosis patients (n=14)**
Trauma with vegetative matter (n=8) Trauma with other objects (n=3) Facial nerve palsy (n=1) Entry of foreign body into the eye (n=1). None (n=2)	Use of occlusive footwear (n=7) Trauma (n=5) None (n=2)

**Table 3:** Minimum inhibitory concentration of *Fusarium *isolates by broth microdilution method

**Species**	**No.**	**MIC mean value and range (g/ml)**					
		**AMB** **(GM/R)**	**AFG** **(GM/R)**	**CAS** **(GM/R)**	**FLC** **(GM/R)**	**VRC** **(GM/R)**	**ITC** **(GM/R)**
Keratitis group							
*Fusarium proliferatum*	7	1.07 0.5 to 2	13.75 0.25 to 16	48.14 1 to 64	41.14 8 to 64	4.07 0.5 to 8	12.28 2 to 16
*Fusarium falciforme*	6	0.6 0.12 to 1	13.67 2 to 16	54 4 to 64	53.67 2 to 64	3.58 0.5 to 8	12.67 4 to 16
*Fusarium keratoplasticum*	6	0.83 0.25 to 1	12.24 2 to 16	42.25 0.5 to 64	45.23 8 to 64	3.73 2 to 8	11.26 4 to 16
*Fusarium incarnatum *SC sp.	6	0.99 0.12 to 4	12.41 0.0625 to 16	51.53 0.5 to 64	50.76 4 to 64	3.73 0.0625 to 8	11.36 1 to 16
*Fusarium sacchari*	5	0.92 0.5 to 1	13.85 16	52.50 16 to 64	64 64	3.99 0.0625 to 16	13.05 4 to 16
*Fusarium solani*	1	0.25 0.25	16 16	16 16	64 64	2 2	16 16
*Fusarium anthophilum*	1	0.12 0.12	16 16	32 32	64 64	4 4	16 16
*Fusarium equiseti *SC sp.	1	0.5 0.5	16 16	64 64	64 64	16 16	16 16

AMB: amphotericin B, AFG: anidulafungin, CAS: caspofungin, FLC: fluconazole, VRC: voriconazole, ITC: itraconazole

## Discussion

Fusariosis is an emerging cause of morbidity and mortality in both developing and developed countries. Given the growth of patients with immunocompromised status, the fraction of at risk population is on an increasing trend making more people prone to the development of this disease. This is consistent with the increased incidence of disseminated *Fusarium* infections in humans, alongside other invasive fungal infections [7, 26]. In this study, the mean age of the patients was 46.2 years (median age: 45 years) with a range of 7-72 years. The frequency of male patients (72.7%) was higher than that of female patients (27.3%), which is in agreement with the epidemiological survey conducted by the European Confederation of Medical Mycology (ECMM) [27].

Though the mean duration of the symptoms reported at the time of presentation among keratitis patients was approximately 22 days, the majority of them presented with severe symptoms. Most of the patients had complaints of redness, pain, watering, photophobia, and diminution of vision in the affected eye. One patient presented with perforated keratitis 2 months after the onset of symptoms. This patient had a history of injury with sugarcane leaf and had taken treatment in the form of natamycin and voriconazole eye drops from local hospitals. Another patient with the diminution of vision in the affected eye to the level of perception of hand movement and finger counting presented to the hospital within 1 month of injury with sugarcane leaf in the affected eye. One patient who developed a full-thickness corneal ulcer with hypopyon after the entry of a foreign body into the eye referred to the hospital 18 days after the incidence of foreign body entry. The affected eye had a marked reduction in vision with the patient just being able to perceive the light in that eye.

In this study, FSSC (43.3%) was identified as the most common species complex, followed by FFSC (40%) and FIESC (16.7%). However, regarding FOSC, no species from this complex was isolated from the patients. These species were correlated with the clinical presentations of the patients. Furthermore, certain species were found to be associated with a particular site of involvement. Additionally, 5/6 (83.3%) isolates of *F. falciforme* and 4/5 (80%) isolates of *F. sacchari* were recovered from the patients with keratitis.

According to Guarro [7], the members of FSSC and FOSC account for approximately 60% and 20% of all human infections, respectively. The remaining 20% of infections are also collectively contributed by various members of other species complexes, such as FIESC, GFSC, FCSC, and FDSC. At the species level, four species were identified from these species complexes that are responsible for most of the human infections. These species included *F. petroliphilum*, *F. keratoplasticum,* and two unnamed phylogenetic species, belonging to FOSC and FDSC complexes.

In the current study, certain important observations were made regarding the distribution of species complexes. In the keratitis group, FSSC was the most common species complex. In addition, *F. falciforme *was the most frequent species isolated from these patients, followed by *F. sacchari* belonging to FFSC. This fungus is a soil saprophyte and a plant pathogen. Certain species of *Fusarium* were more consistently associated with a certain geographical area. Among the patients with keratitis, *F. sacchari* was isolated from four cases all of whom had trauma with the vegetative matter as a risk factor. Out of these four patients, three cases specifically reported trauma with sugarcane leaf (*F. sacchari* is a known pathogen in sugarcane farming). All of these cases were reported from two neighboring districts of Haryana raising suspicion of endemic focus in the region for this fungus [28].

Among onychomycosis patients, the use of occlusive footwear was the most common risk factor [29]. Another risk factor observed in our patients was trauma. The factors that increase the chance of fusarial onychomycosis include repeated micro trauma to the nails, hands and feet moist environment, prolonged exposure to pathogenic fungi, greater work activity, and venous insufficiency [30, 31]. Similar factors were obtained in the present study. In this regard, the use of occlusive footwear in a hot and humid climate leads to the sweating of the feet besides blocking transpiration. In addition, the use of open sandals/footwear or barefoot walk in the fields while working causes repeated nail micro trauma. Most of these patients had a long duration of symptoms ranging from a few months to years, and the predominant reason for seeking treatment was the cosmetic appearance of the affected nails.

Apart from these, four other cases of fusariosis were identified in patients with endophthalmitis/sinusitis and two cases had pulmonary involvement. All fusariosis patients were immunocompetent, except for two cases with pulmonary involvement and one case with endophthalmitis and diabetes.

One 50-year-old diabetic patient, who had undergone cataract surgery in the left eye, developed endophthalmitis 3 days after the surgery. This patient was admitted to the hospital and subjected to a vitreous tap. Direct KOH examination was positive for septate hyphae, and the patient was diagnosed as a case of fusarial endophthalmitis based on culture growth which was identified as *F. incarnatum* SC species (FIESC). Another patient was a 16-year-old male with bilateral nasal polyposis and right pansinusitis, who grew fungus from the right maxillary sinus tissue which was identified as *F. falciforme* (FSSC). No identifiable risk factors were found in this patient.

Two immunocompromised patients presented with pulmonary involvement. The first patient was a 72-year-old male who was a known case of lung cancer and chronic obstructive pulmonary disease presented with shortness of breath, cough, and chest pain. On examination, he was found to have a right-sided pleural effusion. The sputum sample was positive for septate fungal hyphae on direct KOH examination and yielded the growth of *Fusarium *species, which was identified as *F. incarnatum* SC species. The patient was put on fluconazole by the clinician and did not survive despite making the best possible efforts to save him.

The other patient, a 47-year-old male farmer from Haryana as a known case of chronic liver disease and pulmonary hypertension with a past history of tuberculosis, came to the hospital with a history of shortness of breath, cough and abdominal distension. He had a bilateral pleural effusion. Sputum sample of this patient was positive for septate fungal hyphae on direct KOH examination and yielded *Fusarium* isolate that was identified as *F proliferatum* by molecular methods. The patient was on treatment with antitubercular agents before fungal culture came positive. By this time, the patient took discharge from the hospital against medical advice. He was lost on follow-up despite attempts made to trace him.

Among the invasive fungal infections of the pulmonary system, though *Fusarium* lags behind *Aspergillus *in incidence, its outcome is poorer with higher morbidity and mortality. The signs and symptoms of pulmonary *Fusarium* infections are usually non-specific [32]. In a study performed on 20 patients of pulmonary fusariosis by Marom *et al.* [33], 19 patients had clinical signs and symptoms that included shortness of breath, rhonchi, chest pain, and hemoptysis. Fever was present in 15 patients, and 13 patients did not survive 1 month after the establishment of diagnosis.

The MIC was reported for each antifungal agent as no breakpoint has been defined yet for *Fusarium* species in the literature. In this study, the MIC testing was performed for the available antifungals, such as amphotericin B, voriconazole, itraconazole, fluconazole, anidulafungin, and caspofungin, among which amphotericin B was the only drug with low MIC with a mean MIC of 0.74 µg/ml. In a survey conducted by the European Confederation of Medical Mycology to examine invasive fusariosis, the antifungal susceptibility testing of 54 isolates was reported [27]. In the mentioned study, the mean MIC values of amphotericin B, posaconazole, voriconazole, and itraconazole were 1.51, 7.60, 3.01, and 4.16 mg/L, respectively. This is consistent with the results presented in most of the studies in which amphotericin B usually exhibited a lower MIC value in vitro as compared to other drugs [19].

No interpretative MIC breakpoints have been identified for *Fusarium* species yet [17]. Moreover, the in vitro and in vivo correlations may not be present; therefore, the accurate significance of these MICs in clinical practice has not yet been ascertained [34]. Despite the high MIC values seen in vitro, voriconazole appears to be clinically effective [27]. Accordingly, Nucci *et al.* [35] have reported improved outcomes in the patients with invasive fusariosis associated with the use of voriconazole [36].

The keratitis patients responded well and were treated with a combination of various treatment modalities, including surgery, if required, due to inadequate response to the antifungals. The patients were treated with a variety of antifungals, such as oral itraconazole, voriconazole, natamycin, or amphotericin B eye drops and intraconjunctival injection. The patients not responding to these antifungals underwent therapeutic penetrating keratoplasty. Most of the patients with onychomycosis were treated with itraconazole and ciclopirox olamine lacquer. Due to a long period of treatment and follow-up in onychomycosis patients to ascertain the treatment outcome, coupled with high chances of recurrence, we were unable to make a comment on the treatment outcome in these patients.

Lack of epidemiological data regarding *Fasorium* infection in the Northern region of India highlights the significance of the present study. In the current research, a total of 30 cases of fusariosis were diagnosed and identified by molecular techniques in a short period of time (i.e., 18 months) from a single institute. There were a few limitations in this study. Long-term follow-up of the patients to check recurrence, especially in cases with onychomycosis, could not be ascertained. Onychomycosis tends to frequently recur in patients, and it is difficult to completely manage this infection.

A larger study over a long duration with a larger number of isolates is required to better understand the epidemiology of this infection with statistical analysis. In addition, it is required to perform a similar study at the institutes entailing facilities for stem cell and solid organ transplantation to investigate the local epidemiology of invasive fusariosis.

## Conclusion

As the results of the present study indicated, *Fusarium* species are an important cause of infection in the region under investigation in both immunocompetent and immunocompromised individuals. Accurate identification at species level requires molecular methods. However, these methods are not available in the majority of the centers. The advancement in these techniques would result in the modification of the nomenclature and identification of newer species as the causative agents of fusariosis. They show high in vitro MIC to the most commonly used antifungals. Timely diagnosis and appropriate treatment will help to improve the patient outcome.
